# Medical Topics

**Published:** 1890-02

**Authors:** 


Medical Topics, published by W. A. Chatterton, 181 Clark St., Chicago. Price, 25 cents a year.

This is a neat little sixteen-page journal published monthly. The copy before us is the first one ever issued.

Its object is to give the busy practitioner clear and exceedingly concise ideas of all the latest developments in medicine. No long articles are admitted to its pages. Everything is reduced to the dimensions of a paragraph. The idea is to get a "short communication from every physician—only a few lines or words—giving some experience or some fact which will be interesting and useful to some other member of the profession."

The low price places it within the reach of all, and it should accordingly command an immense circulation. W. M. J.

